# Pain at the end of life in patients with cancer: a population-based study on prevalence, relief, and the role of pain assessment

**DOI:** 10.1007/s00520-026-10349-y

**Published:** 2026-01-22

**Authors:** Ellis Slotman, Christel Hedman, Heidi P. Fransen, Yvette M. van der Linden, Natasja J. H. Raijmakers, Staffan Lundström

**Affiliations:** 1 Department of Health Technology and Services Research, University of Twente, Technical Medical Centre, Enschede, the Netherlands; 2 Department of Research and Development, Netherlands Comprehensive Cancer Organisation, Utrecht, the Netherlands; 3 Department of Research and Development, Stockholms Sjukhem Foundation, Stockholm, Sweden; 4 Department of Molecular Medicine and Surgery, Karolinska Institutet, Karolinska University Hospital, Stockholm, Sweden; 5 Centre of Expertise in Palliative Care, Leiden University Medical Centre, Leiden, the Netherlands; 6 Department of Radiotherapy, Leiden University Medical Centre, Leiden, the Netherlands; 7 Department of Oncology-Pathology, Karolinska Institutet, Karolinska University Hospital, Stockholm, Sweden

**Keywords:** Cancer, Pain prevalence, Relief

## Abstract

**Background:**

Pain is common in advanced cancer, and its assessment is recognized as crucial for effective management. However, real-world evidence on pain prevalence, relief, and the impact of structured pain assessment across cancer types at the end of life remains limited.

**Methods:**

We analyzed data from 215,317 patients who died from cancer reported to the Swedish Register of Palliative Care (2011–2023). Data are based on validated end-of-life questionnaires completed by healthcare providers after the patient’s death. Patient characteristics and provider-reported pain outcomes (prevalence of pain, severe pain, structured pain assessment usage, pain relief) were evaluated. Pain prevalence and relief across cancer types were examined through multivariable logistic regression analyses.

**Results:**

Overall, 82% of patients experienced pain and 35% severe pain during their final week of life. Highest pain prevalence occurred in pancreatic, prostate, and bone/soft tissue cancer and lowest in brain/CNS cancers. Complete pain relief was reported in 77% of patients, with lowest odds in patients with prostate and bone/soft tissue cancer and highest odds in patients with brain/CNS cancer. Pain assessment using validated tools was reported in 57% of patients, ranging from 49% in hematological malignancies to 64% in pancreatic cancer. Structured pain assessment was significantly associated with higher odds of complete pain relief both overall (adjusted OR 1.27, 95% CI 1.24–1.30) and across most cancer types.

**Conclusion:**

Pain remains highly prevalent in patients with cancer at the end of life, with variation in both occurrence and relief across cancer types. Structured pain assessment was consistently associated with higher odds of complete pain relief. These findings underscore the importance of routine, systematic pain assessment and tailored pain management strategies in end-of-life cancer care.

**Supplementary Information:**

The online version contains supplementary material available at 10.1007/s00520-026-10349-y.

## Introduction

Pain is one of the most common symptoms in patients with cancer. A recent systematic literature review found pain is prevalent in nearly 45% of patients [1], with the highest rates observed in patients with advanced, metastatic, or terminal disease. More than half (55%) of them experience any pain and up to 40% experience moderate to severe pain. Pain is known to increase toward the end of life [2, 3], and many patients with cancer experience episodes of breakthrough pain on top of otherwise controlled background pain [4].

Experiencing pain significantly diminishes quality of life and increases psychological distress, with many patients citing pain as a primary concern as they approach the end of life [5–8]. Consequently, effective and timely pain management is essential not only for providing emotional comfort and preserving dignity during patients’ final days, but also for preventing unnecessary suffering for both patients and their families. Insight into the prevalence and relief of pain is therefore critical for improving end-of-life care in patients with cancer. Since cancer represents a heterogeneous group of diseases with varying pain mechanisms and treatment strategies, more detailed insight into pain outcomes by cancer type would be valuable to guide patient-centered pain management.

Accurate assessment of symptoms is widely recognized as a critical first step in appropriate pain management and is identified as one of the guiding principles in the cancer pain management guidelines of the World Health Organization (WHO) [9]. Robust evidence from controlled trials has demonstrated that routine symptom assessment improves both symptom control and health-related quality of life in patients with cancer [10–14]. However, more detailed insight into how pain assessment influences pain relief in end-of-life cancer care within routine clinical practice remains limited. This is particularly relevant given the known increase in pain prevalence toward the end of life [2, 3], alongside potentially greater barriers to effective pain assessment. These barriers include communication difficulties, clinician discomfort with end-of-life discussions, and various care settings during end-of-life care (e.g., hospital, specialized palliative care, home), which may lead to inconsistencies in the assessment and management of pain [15–17].

Identifying cancer-specific patterns in pain prevalence and relief at the end of life, along with understanding how structured assessment influences pain outcomes, could inform quality improvement initiatives in palliative oncology care. Therefore, this study aimed to examine pain prevalence and the extent of pain relief during the last week of life across cancer types in a population-based cohort of patients who died from cancer. Additionally, we aimed to assess differences in pain, severe pain, and pain relief according to specialist palliative care involvement, as well as to explore the association between use of structured pain assessment tools and pain relief.

## Methods

### Data source and study cohort

Data was retrieved from the Swedish Register of Palliative Care (SRPC). This registry collects data on end-of-life symptoms and care variables during the last week of patients’ lives using a validated end-of-life questionnaire (ELQ) completed retrospectively after a patient’s death by healthcare professionals, most commonly nurses involved in the patient’s end-of-life care [18, 19]. The registry is intended to capture data on all patients who have died, but as completion of the end-of-life questionnaire is not universal, approximately 60% of all annual deaths and 80% of cancer deaths in Sweden are included in the registry [20, 21]. The SRPC is regularly linked to the Swedish Cause of Death Register, thereby linking the cause of death to all patients included in the SRPC according to the ICD-10 classification. For this study, we included all adult patients who died from cancer (ICD10 codes C00-C96) between 2011 and 2023 and were reported to the SRPC. Only patients with an anticipated death based on their disease trajectory, as indicated in the end-of-life questionnaire, were included in this study.

### Data and definitions

For all included patients, we collected the following characteristics from the SRPC: age, sex, place of death, cause of death, the presence of an end-of-life discussion, and whether there was consultation of external expertise in the management of the patients’ symptoms. Place of death was classified as own home, nursing home permanent stay, nursing home short-term stay, hospital ward (excluding palliative inpatient care), hospice or palliative inpatient care, or other. Cancer types were classified into the categories displayed in Table 1. A further specification of the ICD-10 codes included in these categories can be found in Supplementary Table 1. End-of-life discussion was categorized as present or absent based on whether the patient received information about the transition to end-of-life care. Consultation of external expertise was categorized as yes or no based on whether at least one of the following specialists outside the care team was consulted about the patient’s symptoms during the last week of life: pain clinic, palliative care team, other hospital unit, spiritual counsellor, or allied health professionals.

**Table 1:** Cohort characteristics

	***N***	**%**
Total number of patients	215,317	
**Characteristics**		
Age (median; interquartile range)	76	(68–84)
Sex		
* Male*	110,244	51
* Female*	105,073	49
Place of death		
* Hospice or palliative inpatient care*	66,792	31
* Own home*	53,657	25
* Hospital ward*	47,836	22
* Nursing home—permanent stay*	23,215	11
* Nursing home—short-term stay*	22,818	11
* Other*	999	1
Communication capacity		
* Able to express will always or until days/hours before death*	191,701	89
* Ability to express will was lost weeks or months before death*	18,420	9
* Don’t know*	5196	2
Cancer type		
* Lung cancer*	33,919	16
* Colorectal cancer*	26,871	13
* Prostate cancer*	21,712	10
* Pancreatic cancer*	19,045	9
* Hematological malignancy*	16,811	8
* Breast cancer*	13,813	6
* Urinary tract cancer*	12,662	6
* Cancer of female genital organs*	12,285	6
* Gastric or esophageal cancer*	10,303	5
* Cancer of unknown primary*	9749	5
* Liver cancer*	7053	3
* Cancer of the brain/CNS*	6149	3
* Skin cancer*	5952	3
* Cancer of the gallbladder/biliary tract*	5083	2
* Cancer of the head and neck region*	4481	2
* Cancer of the bone/soft tissue*	2360	1
* Other*	7069	3

The following variables related to pain prevalence and relief were extracted from the SRPC: use of structured pain assessment tools during the last week of life, occurrence of pain and severe pain, and the extent of pain relief achieved. Pain assessment was based on the question: “Was the person’s pain assessed at any time during the last week of life using VAS, NRS or another pain-assessment tool?” This includes the use of proxy assessment tools, such as the IPOS proxy version and Abbey pain scale, in cases where patients were unable to express themselves. Data on the occurrence of pain were collected through the question: “Did the person display breakthrough of any of the following symptoms during the last week of life?”, with pain listed as one of the symptoms. In this question, breakthrough of symptoms refers to the presence of symptoms on any occasion during the last week of life. Information on severe pain was collected between 2011 and 2021 using the question: “Did the person experience severe pain at any time during the last week of life (e.g., VAS or NRS > 6 or severe pain according to another validated tool)?” The questions regarding pain assessment, occurrence of pain, and occurrence of severe pain included three response options: “yes,” “no,” or “don’t know.” “Don’t know” responses were excluded from all analyses. Pain relief data were collected only for patients with reported pain. The question was “Pain was relieved,” with response options “completely,” “partially,” or “not at all.” In this context, relief was determined by the subjective assessment of the healthcare professional completing the questionnaire. Since the “not at all” category represented < 1% of responses, this category was combined with “partially” for analytical purposes.

### Statistical analyses

Descriptive statistics were used to present the prevalence of pain, severe pain, and the extent of pain relief stratified by cancer type. To evaluate differences in pain experiences between cancer types, logistic regression models were created with prevalence of pain, severe pain, and pain relief as dependent variables and cancer type as the independent variable. The models for prevalence of pain and severe pain were adjusted for age, sex, and place of death. The model for pain relief was additionally adjusted for the presence of an end-of-life discussion and consultation of external expertise in the management of symptoms. Deviation coding was applied to the independent variable to ensure that the association of each cancer type with pain outcomes was assessed relative to the grand mean across all cancer types rather than a single reference category. Due to the use of deviation coding, odds ratios were not provided for the last category (other), as this group is omitted to ensure that the model parameters sum to zero and comparisons are made relative to the grand mean. To evaluate differences in pain, severe pain, and pain relief according to specialist palliative care involvement, we conducted both descriptive analyses and multivariable logistic regression adjusted for age and sex. Analyses were stratified into three groups: (1) patients without specialist palliative care involvement, (2) patients who died in a palliative care setting (hospice or inpatient palliative care) or received specialized palliative home care, and (3) patients who died elsewhere but received specialist palliative care (SPC) consultation for symptom management. To assess the association between pain assessment and the extent of pain relief, logistic regression analyses were performed for the total cohort and for all cancer types separately. These models included complete pain relief as the dependent variable and pain assessment as the independent variable, adjusted for age, sex, place of death, the presence of an end-of-life discussion, and consultation of external expertise in the management of symptoms.

## Results

### Characteristics of the study population

A total of 215,317 patients who died of cancer were included in this population-based cohort study (2011–2023). The median time between the death of a patient and registration in the SRPC ranged from 3 to 7 days during the study period. The median age of patients was 76 years, and a slight majority were male (51%). Specialized palliative care settings were the most common place of death, with 31% of patients dying in hospice or palliative inpatient facilities, followed by home (25%) and general hospital ward (22%). In 9% of patients, it was reported that they lost their ability to express their will weeks or months before death. The most common cancer types were lung cancer (16%), followed by colorectal cancer (13%) and prostate cancer (10%), as detailed in Table 1.

### Prevalence of pain and severe pain at the end of life

**Fig. 1:**
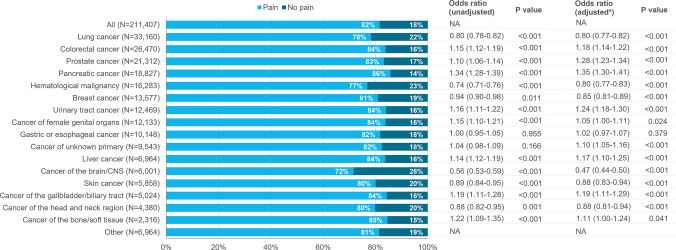
Prevalence (%) and odds ratios for pain during the last week of life in patients with cancer. *Odds ratios were adjusted for age, sex, and place of death. Odds ratios were assessed relative to the grand mean

**Fig. 2:**
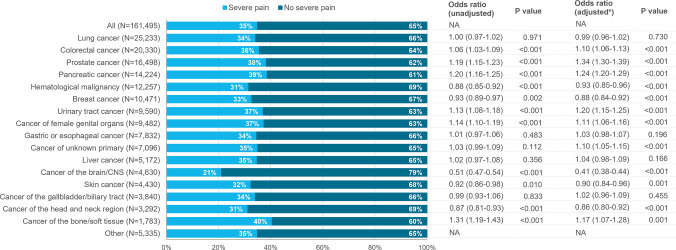
Prevalence (%) and odds ratios for severe pain (pain score > 6) during the last week of life in patients with cancer. *Odds ratios were adjusted for age, sex, and place of death. Odds ratios were assessed relative to the grand mean

Pain was reported in 82% of all patients during their final week of life, with the lowest prevalence in those with brain/CNS cancers (72%) and the highest in those with pancreatic cancer (86%) and cancer of the bone/soft tissue (85%) (Fig. 1). Severe pain was reported in 35% of all patients, also being least common in brain/CNS cancers (21%) and most common in bone/soft tissue cancers (40%) and pancreatic cancer (39%) (Fig. 2). When adjusted for age, sex, and place of death, those with brain/CNS cancers had the lowest odds of both pain (OR 0.47, 95% CI 0.44–0.50) and severe pain (OR 0.41, 95% CI 0.38–0.44) (Figs. 1 and 2). The adjusted odds ratios were highest in patients with pancreatic cancer, with ORs of 1.35 (95% CI 1.30–1.41) for pain and 1.24 (95% CI 1.20–1.29) for severe pain, and patients with prostate cancer, with ORs of 1.28 (95% CI 1.23–1.34) for pain and 1.34 (95% CI 1.30–1.39) for severe pain. In a sensitivity analysis limited to patients whose pain was assessed using a validated tool, the prevalence of severe pain was 5–10% higher across cancer types (Supplementary Figure 1).

### Pain relief

**Fig. 3:**
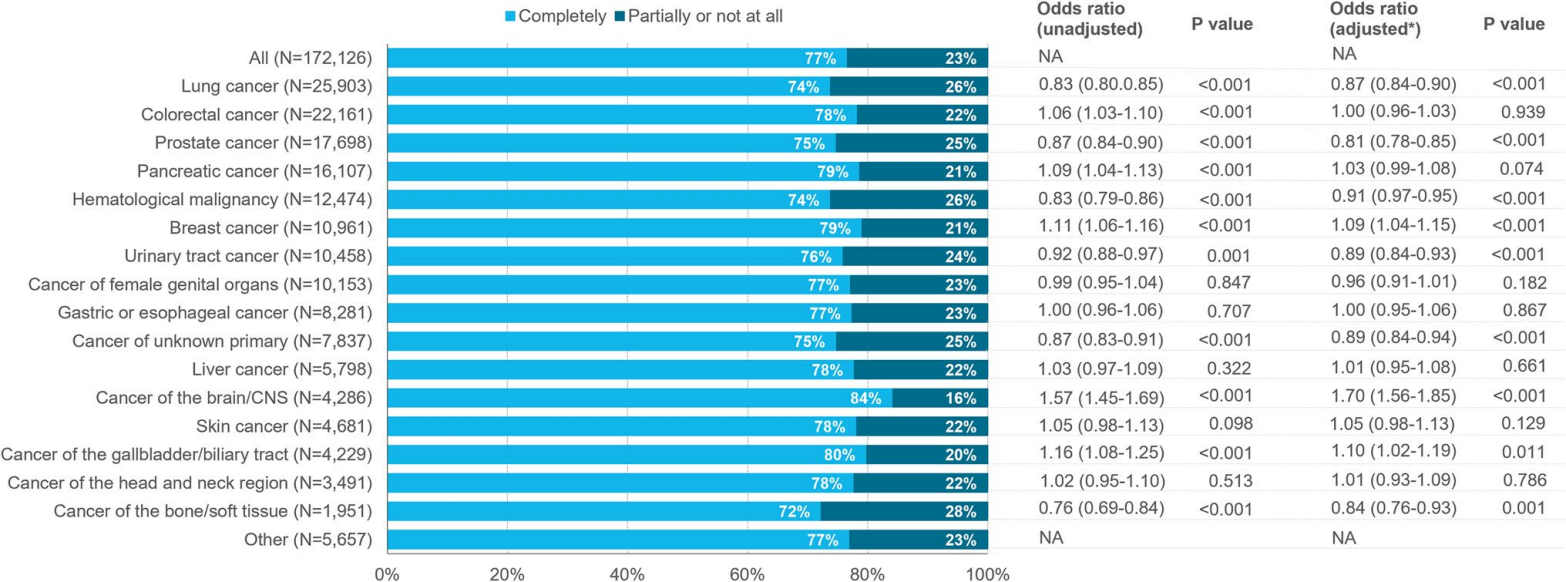
Extent of pain relief and odds ratios for complete pain relief during the last week of life in patients with cancer. *Odds ratios were adjusted for age, sex, place of death, presence of an end-of-life discussion, and consultation of external expertise in the management of symptoms. Odds ratios were assessed relative to the grand mean

Pain relief data was available for 161,495 (94%) of the 172,126 patients experiencing pain during the last week of life. Complete pain relief was reported in 77% of these patients, with complete pain relief being lowest in patients with cancers of the bone/soft tissue (72%) and highest in those with cancer of the brain/CNS (84%) (Fig. 3). Multivariable regression analyses showed lower adjusted odds ratios for complete pain relief in patients with prostate cancer (OR 0.81, 95% CI 0.78–0.85) and cancer of the bone/soft tissue (OR 0.84, 95% CI 0.76–0.93) and higher odds of complete pain relief in patients with cancer of the brain/CNS (OR 1.70, 95% CI 1.56–1.85).

### Palliative care involvement

In the total cohort, specialist palliative care was involved during the last week of life in 134,046 patients (62%), of whom 106,068 died in a palliative care (PC) setting or received specialized palliative home care and 27,978 died elsewhere but had a specialist palliative care consultation for symptom management (Table 2). Pain prevalence was higher among patients with specialist palliative care involvement compared to those without, particularly in patients who did not die in a palliative care setting but received specialist palliative care consultation for symptom management (86% vs. 79%; adjusted OR 1.60, 95% CI 1.54–1.67). Severe pain was also more common in these patients (44% vs. 33%; adjusted OR 1.48, 95% CI 1.44–1.53). Complete pain relief was more likely in patients who died in a palliative care setting or received specialized palliative home care (81% vs. 72%; adjusted OR 1.98, 95% CI 1.93–2.03). These trends were consistently observed across all cancer types (Supplementary Table 2).

**Table 2:** Prevalence of pain, severe pain, and complete pain relief stratified by palliative care involvement

Specialist palliative care involvement	Pain		Severe pain		Complete pain relief	
	%	aOR* (95% CI)	%	aOR* (95% CI)	%	aOR* (95% CI)
No specialist palliative care involvement (*n* = 81,271)	79	ref	33	ref	72	ref
Died in a PC setting^±^ or received specialized palliative home care (*n* = 106,068)	82	1.14 (1.12–1.17)	34	0.93 (0.90–0.95)	81	1.98 (1.93–2.03)
Died elsewhere but had SPC^¥^ consultation for symptom management (*n* = 27,978)	86	1.60 (1.54–1.67)	44	1.48 (1.44–1.53)	70	1.00 (0.98–1.04)

*Odds ratios were adjusted for age and sex

^±^PC (palliative care) setting includes hospice and inpatient palliative care

^¥^*SPC* specialist palliative care

### Pain assessment and its association with pain relief

**Fig. 4:**
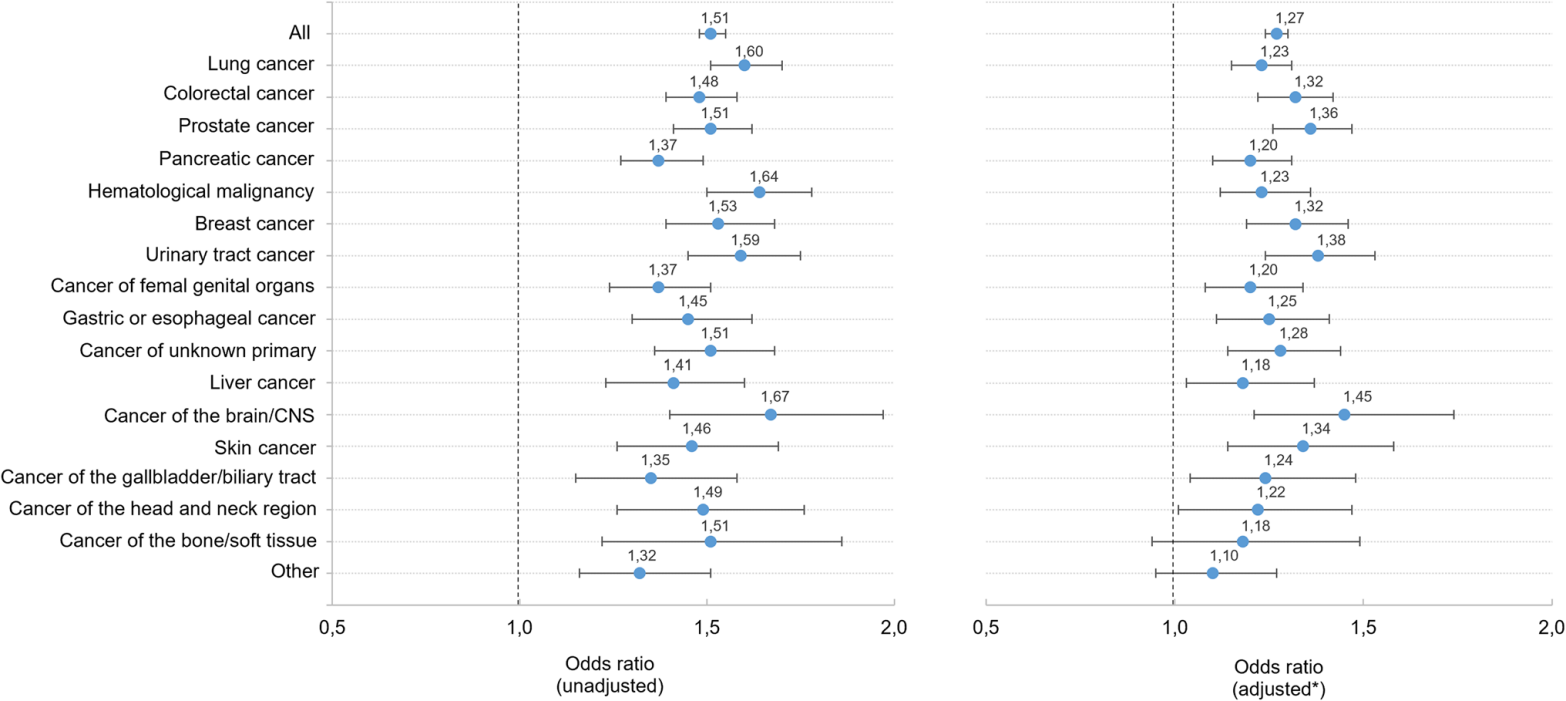
Odds ratios for the association between pain assessment (VAS, NRS, or other validated tool) and complete pain relief during the last week of life in patients with cancer. *Odds ratios were adjusted for age, sex, place of death, presence of an end-of-life discussion, and consultation of external expertise in the management of symptoms

Use of structured pain assessment using validated tools (VAS, NRS, or equivalent) was reported in 57% of all patients during the last week of life. It was least frequently performed in patients with hematological malignancies (49%) and most frequently in those with pancreatic cancer (64%). Univariable regression analyses showed that patients whose pain was assessed during the last week of life had higher odds of achieving complete pain relief (OR 1.51, 95% CI 1.48–1.55), an association observed consistently across all cancer types (Fig. 4). After adjustment for potential confounders (age, sex, place of death, end-of-life conversation, and external expertise consultation), pain assessment remained significantly associated with higher odds of complete pain relief in most cancer types. The strongest association was observed in patients with brain/CNS cancer (adjusted OR 1.45, 95% CI 1.21–1.74).

## Discussion

In this large population-based cohort study encompassing over 200,000 patients who died from cancer, we investigated pain prevalence and relief at the end of life across diverse cancer diagnoses, with particular focus on the association between structured pain assessment and pain outcomes. Our findings show that pain at the end of life remains highly prevalent in patients with cancer, with some variability in the prevalence and relief of pain across cancer types. Importantly, use of structured pain assessment was consistently associated with a higher likelihood of achieving complete pain relief among those experiencing pain during the last week of life.

Overall, pain during the last week of life was reported in 82% of patients, and approximately one-third experienced severe pain. These findings point to a substantial pain burden among patients dying from cancer, which is consistent with previous studies demonstrating that pain is highly prevalent in patients with advanced or terminal cancer, particularly toward the end of life [1–3, 22]. There was some variability in the prevalence of pain across cancer types, with pain being most prevalent in patients with pancreatic, prostate, and bone or soft tissue cancers. This may be attributed to the complexity of pain in these patients, involving multiple pain mechanisms. In pancreatic cancer, patients often suffer from severe abdominal pain that can be visceral and somatic as well as neuropathic in nature, caused by ductal obstruction, tissue damage, or invasion or compression of nerves [23–25]. In patients with advanced prostate cancer, which has a high rate of metastasis to the bone, and in bone or soft tissue cancers, inflammatory, mechanical, and neuropathic elements of pain are likely to be present due to bone invasion and destruction [26, 27]. Complete pain relief was also less frequently achieved in patients with bone and soft tissue cancers, and to some extent in those with prostate cancer, which may reflect this inherent complexity of the underlying pain mechanisms. In contrast, patients with brain or central nervous system (CNS) tumors had a lower prevalence of pain and a higher likelihood of complete pain relief. Due to the absence of pain receptors in the brain, brain tumors do not cause pain directly. Additionally, because these tumors typically do not spread beyond the nervous system, patients usually do not experience pain from metastases in other organs. Pain in these patients is most commonly related to increased intracranial pressure [28, 29], which may be effectively relieved with corticosteroids or other targeted agents aimed at reducing inflammation and edema [30, 31].

This study also underscores the role of palliative care in managing pain at the end of life. Notably, patients who died in a palliative care setting (hospice, inpatient palliative care unit, specialized palliative home care) were more likely to achieve complete pain relief, despite experiencing slightly higher rates of pain. This finding may reflect the specialized education and adherence to palliative care principles among healthcare providers in these settings, highlighting the value of extending such training to all professionals involved in cancer care. Furthermore, a consistently higher rate of severe pain was observed among patients who died outside of palliative care settings but received specialist palliative care consultations for symptom management. This likely reflects the greater complexity and severity of pain in these cases, which prompted the need for specialist input. Although severe pain was more common in this group, their likelihood of complete pain relief matched that of patients without specialist palliative care involvement, underscoring the value of specialist input for managing severe pain.

Moreover, structured pain assessment during the last week of life was significantly associated with a higher likelihood of complete pain relief across cancer types. This robust finding supports and extends the benefits of routine symptom assessment, previously demonstrated in controlled clinical trial settings, to real-world end-of-life care. Several clinical trials have demonstrated that standardized symptom assessment improves clinical outcomes in patients with cancer [10–14]. For example, the implementation of the structured use of the Edinburgh Pain Assessment and Management Tool improved pain documentation and management in inpatient settings [10]. A trial by Basch et al. showed that electronic symptom monitoring enhanced symptom control and quality of life during cancer treatment [11, 12]. Similarly, the SYMPRO-Lung trial found that patient-initiated symptom monitoring led to timely clinical responses and improved quality of life in patients with lung cancer [14]. The results of this present study extend this evidence to the context of pain management in end-of-life cancer care in routine clinical practice, suggesting that pain assessments can significantly improve pain relief in patients with cancer at the end of life. Several factors may explain this association. Routine assessment of pain may improve communication about and awareness and documentation of pain [32–34]. Furthermore, it may prompt more timely clinical action and may facilitate earlier escalation or adjustment of pain treatments [32, 35].

The strength of the association between pain assessment and complete pain relief showed some variation across cancer types, likely reflecting multiple contributing factors. First, the intrinsic nature and complexity of pain associated with each cancer type can influence the degree to which pain is responsive to treatment, even when it is routinely assessed. In addition, assessment alone is not sufficient to improve outcomes, and correct and timely clinical responses are essential to achieving effective pain relief [36]. Therefore, differences in how healthcare professionals respond to pain assessments may also contribute to how well pain is managed. For example, studies have shown that oncologists generally have better knowledge about cancer pain management compared to other healthcare providers such as nurses, general practitioners, internal medicine specialists, or surgeons [15, 37, 38]. Since the level of involvement of different specialists can vary depending on the type of cancer, this variation in knowledge of and experience with managing cancer-specific pain may contribute to differences in treatment effectiveness. Additionally, variations in the adherence to evidence-based pain management guidelines may influence the degree of pain relief achieved following assessment [39].

The findings of this study highlight the crucial role of effective pain management in ensuring high-quality end-of-life care for patients with cancer, given the high prevalence of pain across all cancer types. Within this context, the results of this study also underscore the importance of pain assessments in effective pain management, as these were associated with improved pain relief. Although existing pain management guidelines emphasize the important role of routine pain assessment [9, 40], adherence to pain assessment is still suboptimal and mentioned as one of the main barriers to effective pain management [15–17, 41, 42]. Improving the consistent use of pain assessments therefore represents an opportunity for optimizing pain management for patients dying from cancer. In the Netherlands, national norms for oncologists now mandate routine symptom assessment in all patients with cancer with a life expectancy shorter than a year [43]. Such initiatives that go beyond emphasizing the importance of symptom assessment by framing it as a requirement for high-quality oncology care may help ensure its routine implementation and thereby contribute to improved pain management. Furthermore, integrating education on pain mechanisms and treatment strategies into the training of all healthcare professionals involved in cancer care may help ensure that pain assessments are consistently followed by adequate and tailored pain management strategies.

### Strengths and limitations

The main strength of this large cohort study is the inclusion of data from a nation-wide cohort of over 200,000 patients dying from cancer across different healthcare settings, thereby giving a good representation of daily clinical practice. However, several limitations should be noted. First, the quality of the data in the SRPC is inherently dependent on documentation by the healthcare provider, potentially introducing variability in accuracy and completeness. Additionally, the question on pain relief specifically is somewhat subjective, and interpretations of “complete” or “partial” relief may differ. Furthermore, since responses indicating incomplete pain relief (partially or not at all) could be perceived as a reflection of inadequate care, there is a possibility that these answers are underreported. Another limitation is that, in cases where patients are unable to express themselves due to cognitive impairment or delirium, the prevalence and severity of pain have to be assessed using proxy measures. The use of proxy assessments may introduce bias, as these tools rely on the interpretation of healthcare providers rather than direct patient reporting. Moreover, because the questions on pain prevalence and severity refer to whether these symptoms were present at any time during the last week of life, and there is no question about the localization of pain, the data cannot capture the full complexity of pain experiences at the end of life. Additionally, due to the retrospective and observational nature of this study, causality between pain assessment and pain relief cannot be established and not all potential confounding variables could be accounted for. Although it seems less likely that improved pain relief leads to more assessments, and the analyses were adjusted for key variables that may influence both pain assessment and pain relief, these findings should still be interpreted with caution.

## Conclusion

This large population-based cohort study showed a substantial pain burden among patients with cancer during their final week of life, with 82% experiencing pain and 35% suffering from severe pain. Some variation in both pain prevalence and relief was observed across cancer types. Importantly, dying in a palliative care setting and structured pain assessment were consistently associated with improved pain relief across cancer types. These findings highlight the critical importance of individualized pain management strategies, palliative care education, and structured pain assessment in optimizing end-of-life cancer care quality.

## Supplementary Information

Below is the link to the electronic supplementary material.

Supplementary file1 (DOCX 96 KB)

## Data Availability

The datasets generated, used, and analyzed during the current study is available from the corresponding author on reasonable request.

## References

[1] Snijders RAH, Brom L, Theunissen M, van den Beuken- Everdingen MHJ (2023) Update on prevalence of pain in patients with cancer 2022: a systematic literature review and meta-analysis. Cancers (Basel). 10.3390/cancers15030591 36765547 10.3390/cancers15030591 PMC9913127

[2] Smith AK, Cenzer IS, Knight SJ et al (2010) The epidemiology of pain during the last 2 years of life. Ann Intern Med 153(9):563–569. 10.7326/0003-4819-153-9-201011020-00005 21041575 10.1059/0003-4819-153-9-201011020-00005 PMC3150170

[3] Chaudhry I, Shafiq M, Teo I, Ozdemir S, Malhotra C (2022) Epidemiology of pain among patients with solid metastatic cancer during the last year of life. J Pain Res. 10.2147/jpr.s375874 36147456 10.2147/JPR.S375874 PMC9488613

[4] Deandrea S, Corli O, Consonni D, Villani W, Greco MT, Apolone G (2014) Prevalence of breakthrough cancer pain: a systematic review and a pooled analysis of published literature. J Pain Symptom Manage 47(1):57–76. 10.1016/j.jpainsymman.2013.02.015 23796584 10.1016/j.jpainsymman.2013.02.015

[5] Costantini M, Ripamonti C, Beccaro M et al (2009) Prevalence, distress, management, and relief of pain during the last 3 months of cancer patients’ life. Results of an Italian mortality follow-back survey. Ann Oncol 20(4):729–735. 10.1093/annonc/mdn700 19164455 10.1093/annonc/mdn700

[6] Mystakidou K, Tsilika E, Parpa E, Katsouda E, Galanos A, Vlahos L (2006) Psychological distress of patients with advanced cancer: influence and contribution of pain severity and pain interference. Cancer Nurs 29(5):400–405. 10.1097/00002820-200609000-00009 17006114 10.1097/00002820-200609000-00009

[7] Gonella S, Sperlinga R, Sciannameo V, Dimonte V, Campagna S (2019) Characteristics of breakthrough pain and its impact on quality of life in terminally ill cancer patients. Integr Cancer Ther 18:1534735419859095. 10.1177/1534735419859095 31220961 10.1177/1534735419859095 PMC6589949

[8] Hjermstad MJ, Kaasa S, Caraceni A et al (2016) Characteristics of breakthrough cancer pain and its influence on quality of life in an international cohort of patients with cancer. BMJ Support Palliat Care 6(3):344–352. 10.1136/bmjspcare-2015-000887 27342412 10.1136/bmjspcare-2015-000887

[9] Organization WH (2018) WHO guidelines for the pharmacological and radiotherapeutic management of cancer pain in adults and adolescents. World Health Organization 30776210

[10] Fallon M, Walker J, Colvin L, Rodriguez A, Murray G, Sharpe M (2018) Pain management in cancer center inpatients: a cluster randomized trial to evaluate a systematic integrated approach-the Edinburgh pain assessment and management tool. J Clin Oncol 36(13):1284–1290. 10.1200/jco.2017.76.1825 29543567 10.1200/JCO.2017.76.1825 PMC5929219

[11] Basch E, Schrag D, Henson S et al (2022) Effect of electronic symptom monitoring on patient-reported outcomes among patients with metastatic cancer: a randomized clinical trial. JAMA 327(24):2413–2422 35661856 10.1001/jama.2022.9265 PMC9168923

[12] Basch E, Deal AM, Kris MG et al (2015) Symptom monitoring with patient-reported outcomes during routine cancer treatment: a randomized controlled trial. J Clin Oncol 34(6):557–565. 10.1200/JCO.2015.63.0830 10.1200/JCO.2015.63.0830 PMC4872028 26644527

[13] Adam R, Burton CD, Bond CM, De Bruin M, Murchie P (2017) Can patient-reported measurements of pain be used to improve cancer pain management? A systematic review and meta-analysis. BMJ Support Palliat Care 7(4):00–00 10.1136/bmjspcare-2016-001137 27879472

[14] Billingy NE, van den Hurk CJ, Tromp VN et al (2024) Patient-vs physician-initiated response to symptom monitoring and health-related quality of life: the SYMPRO-Lung Cluster Randomized Trial. JAMA Netw Open 7(8):e2428975–e2428975 39186274 10.1001/jamanetworkopen.2024.28975

[15] Kwon JH (2014) Overcoming barriers in cancer pain management. J Clin Oncol 32(16):1727–1733 24799490 10.1200/JCO.2013.52.4827

[16] Jacobsen R, Liubarskienë Z, Møldrup C, Christrup L, Sjøgren P, Samsanavičienë J (2009) Barriers to cancer pain management: a review of empirical research. Medicina 45(6):427 19605961

[17] Oldenmenger WH, Sillevis Smitt PA, van Dooren S, Stoter G, van der Rijt CC (2009) A systematic review on barriers hindering adequate cancer pain management and interventions to reduce them: a critical appraisal. Eur J Cancer 45(8):1370–1380. 10.1016/j.ejca.2009.01.007 19201599 10.1016/j.ejca.2009.01.007

[18] Martinsson L, Heedman PA, Lundström S, Fransson G, Axelsson B (2011) Validation study of an end-of-life questionnaire from the Swedish register of palliative care. Acta Oncol 50(5):642–647. 10.3109/0284186x.2011.554434 21391772 10.3109/0284186X.2011.554434

[19] Martinsson L, Heedman PA, Lundström S, Axelsson B (2017) Improved data validity in the Swedish register of palliative care. PLoS One 12(10):e0186804. 10.1371/journal.pone.0186804 29049396 10.1371/journal.pone.0186804 PMC5648220

[20] Martinsson L, Strang P, Lundström S, Hedman C (2024) Parenteral hydration in dying patients with cancer: a national registry study. J Pain Symptom Manage 67(5):384–392. 10.1016/j.jpainsymman.2024.01.036 38342476 10.1016/j.jpainsymman.2024.01.036

[21] Svenska Palliativ Registret (2023) Årsrapport för Svenska palliativregistret 2023 [Annual report for the Swedish Registry of Palliative Care 2023]. Accessed April 23 2025. https://www.palliativregistret.se/arkiv/aarsrapporter/

[22] Hedman C, Fürst P, Strang P, Schelin ME, Lundström S, Martinsson L (2024) Pain prevalence and pain relief in end-of-life care–a national registry study. BMC Palliat Care 23(1):171 39004730 10.1186/s12904-024-01497-1 PMC11247729

[23] Koulouris AI, Banim P, Hart AR (2017) Pain in patients with pancreatic cancer: prevalence, mechanisms, management and future developments. Dig Dis Sci 62:861–870 28229252 10.1007/s10620-017-4488-z

[24] Lahoud MJ, Kourie HR, Antoun J, El Osta L, Ghosn M (2016) Road map for pain management in pancreatic cancer: a review. World J Gastrointest Oncol 8(8):599 27574552 10.4251/wjgo.v8.i8.599 PMC4980650

[25] Lohse I, Brothers SP (2020) Pathogenesis and treatment of pancreatic cancer related pain. Anticancer Res 40(4):1789–1796 32234867 10.21873/anticanres.14133 PMC7323503

[26] Zajączkowska R, Kocot-Kępska M, Leppert W, Wordliczek J (2019) Bone pain in cancer patients: mechanisms and current treatment. Int J Mol Sci 20(23):6047 31801267 10.3390/ijms20236047 PMC6928918

[27] Falk S, Dickenson AH (2014) Pain and nociception: mechanisms of cancer-induced bone pain. J Clin Oncol 32(16):1647–1654 24799469 10.1200/JCO.2013.51.7219

[28] Walbert T, Khan M (2014) End-of-life symptoms and care in patients with primary malignant brain tumors: a systematic literature review. J Neurooncol 117:217–224 24522718 10.1007/s11060-014-1393-6

[29] Koekkoek JA, van der Meer PB, Pace A et al (2023) Palliative care and end-of-life care in adults with malignant brain tumors. Neuro Oncol 25(3):447–456 36271873 10.1093/neuonc/noac216 PMC10013651

[30] Ryan R, Booth S, Price S (2012) Corticosteroid-use in primary and secondary brain tumour patients: a review. J Neurooncol 106:449–459 21971734 10.1007/s11060-011-0713-3

[31] Kaal EC, Vecht CJ (2004) The management of brain edema in brain tumors. Curr Opin Oncol 16(6):593–600 15627023 10.1097/01.cco.0000142076.52721.b3

[32] Howell D, Molloy S, Wilkinson K et al (2015) Patient-reported outcomes in routine cancer clinical practice: a scoping review of use, impact on health outcomes, and implementation factors. Ann Oncol 26(9):1846–1858. 10.1093/annonc/mdv181 25888610 10.1093/annonc/mdv181

[33] Detmar SB, Muller MJ, Schornagel JH, Wever LD, Aaronson NK (2002) Health-related quality-of-life assessments and patient-physician communication: a randomized controlled trial. JAMA 288(23):3027–3034 12479768 10.1001/jama.288.23.3027

[34] Bainbridge D, Seow H, Sussman J et al (2011) Multidisciplinary health care professionals’ perceptions of the use and utility of a symptom assessment system for oncology patients. J Oncol Pract 7(1):19–23 21532805 10.1200/JOP.2010.000015 PMC3014504

[35] Seow H, Sussman J, Martelli-Reid L, Pond G, Bainbridge D (2012) Do high symptom scores trigger clinical actions? An audit after implementing electronic symptom screening. J Oncol Pract 8(6):e142–e148 23598849 10.1200/JOP.2011.000525 PMC3500488

[36] Rosenbloom SK, Victorson DE, Hahn EA, Peterman AH, Cella D (2007) Assessment is not enough: a randomized controlled trial of the effects of HRQL assessment on quality of life and satisfaction in oncology clinical practice. Psychooncology 16(12):1069–1079 17342789 10.1002/pon.1184

[37] Makhlouf SM, Pini S, Ahmed S, Bennett MI (2020) Managing pain in people with cancer-a systematic review of the attitudes and knowledge of professionals, patients, caregivers and public. J Cancer Educ 35(2):214–240. 10.1007/s13187-019-01548-9 31119708 10.1007/s13187-019-01548-9 PMC7076060

[38] Ayoub NM, Jibreel M, Nuseir K, Al-Taani GM (2022) A survey of knowledge and barriers of healthcare professionals toward opioid analgesics in cancer pain management. Int J Clin Pract 2022(1):1136430. 10.1155/2022/1136430 35685510 10.1155/2022/1136430 PMC9159223

[39] Mearis M, Shega JW, Knoebel RW (2014) Does adherence to National Comprehensive Cancer Network guidelines improve pain-related outcomes? An evaluation of inpatient cancer pain management at an academic medical center. J Pain Symptom Manage 48(3):451–458. 10.1016/j.jpainsymman.2013.09.016 24439844 10.1016/j.jpainsymman.2013.09.016

[40] Fallon M, Giusti R, Aielli F et al (2018) Management of cancer pain in adult patients: ESMO clinical practice guidelines. Ann Oncol 29:iv166–iv191. 10.1093/annonc/mdy152 30052758 10.1093/annonc/mdy152

[41] Besse K, Steegers M, Vernooij-Dassen M, Vissers K, Engels Y (2017) Dutch pain specialists’ adherence to the multidisciplinary guideline on treating pain in patients with cancer: a case vignette study. Pain Pract 17(3):344–352. 10.1111/papr.12453 27106621 10.1111/papr.12453

[42] Te Boveldt N, Vernooij-Dassen M, Besse K, Vissers K, Engels Y (2015) Adoptation of an evidence-based clinical practice guideline in cancer pain management by medical oncologists: a case vignette study. Support Care Cancer 23:1409–1420 25370888 10.1007/s00520-014-2472-0

[43] Federatie Medisch Specialisten (2025) SONCOS normeringsrapport 13 2025: Multidisciplinaire normering oncologische zorg in Nederland [SONCOS standardization report 13 2025: Multi-disciplinary standardisation of oncology care in the Netherlands]. https://demedischspecialist.nl/sites/default/files/202502/soncos_normeringsrapport_versie_13_2025.pdf

