# Supplementary material for: Pain at the end of life in patients with cancer: a population-based study on prevalence, relief, and the role of pain assessment
Source: Support Care Cancer. 2026 Jan 22;34(2):116. doi: 10.1007/s00520-026-10349-y (PMC12827307; doi:10.1007/s00520-026-10349-y)
Supplement: Supplementary file 1 — Supplementary file1 (DOCX 96 KB) [file 520_2026_10349_MOESM1_ESM.docx]

# Supplementary material

*Supplementary table 1: Categorization of cancer types based on ICD-10 codes.*

| **Cancer types** | **ICD10-codes** | **Includes** |
| --- | --- | --- |
| Lung cancer | C34 | Malignant neoplasm of bronchus and lung |
| Colorectal cancer | C18,C19,C20 | Malignant neoplasm of colon, rectosigmoid junction and rectum |
| Prostate cancer | C61 | Malignant neoplasms of prostate |
| Pancreatic cancer | C25 | Malignant neoplasms of pancreas |
| Hematological malignancy | C81-C96 | lymphomas, malignant immunoproliferative diseases, leukemia and other and unspecified malignant neoplasms of lymphoid, hematopoietic and related tissues |
| Breast cancer | C50 | Malignant neoplasm of breast |
| Urinary tract cancer | C64-C68 | Malignant neoplasms of kidney, renal pelvis, ureter, bladder and other unspecified urinary organs |
| Cancer of the female genital organs | C51-C57 | Malignant neoplasms of vulva, vagina, cervix, uterus, ovary or other unspecified genital organs |
| Gastric or esophageal cancer | C15,C16 | Malignant neoplasms of stomach or esophagus |
| Cancer of unknown primary | C80 | Malignant neoplasms primary site unknown or unspecified |
| Liver cancer | C22 | Malignant neoplasms of liver and intrahepatic bile ducts |
| Cancer of the brain and central nervous system (CNS) | C70-C72 | Malignant neoplasms of meninges, brain, spinal cord, cranial nerves and other parts of the central nervous system |
| Skin cancer | C43,C44 | Melanoma and other malignant neoplasms of skin |
| Cancer of the gallbladder and biliary tract | C23,C24 | Malignant neoplasms of gallbladder and other unspecified parts of biliary tract |
| Cancer of the head and neck region | C00-C14, C30-C32, C73 | Malignant neoplasms of lip, oral cavity, pharynx, nasal cavity, middle ear, sinuses, larynx and thyroid gland |
| Cancer of the bone and soft tissue | C40,C41,C46,C47,C48,C49 | Malignant neoplasms of bone and articular cartilage, peripheral nerves and autonomic nervous system, retroperitoneum and peritoneum, Kaposi sarcoma, and malignant neoplasms of other connective and soft tissue |
| Other | C17,C26,C33,C37-C39,C45,C58,C60,C62,C63,C69,C74-C76 | Mesothelioma, malignant neoplasms of small intestines, anus and anal canal, trachea, thymus, placenta, penis, testis, eye and adnexa, adrenal gland, and other and unspecified sites |

*Supplementary table 1: Categorization of cancer types based on ICD-10 codes.*

*Supplementary table 2: Prevalence of pain, severe pain and complete pain relief stratified by palliative care involvement and cancer type.*

|  | | **Pain** | | **Severe pain** | | **Complete pain relief** | |
| --- | --- | --- | --- | --- | --- | --- | --- |
|  |  | **%** | **aOR* (95%CI)** | **%** | **aOR* (95%CI)** | **%** | **aOR* (95%CI)** |
| Lung cancer | No specialist palliative care involvement | 76% | ref | 33% | ref | 67% | ref |
|  | Died in a PC^±^ setting or received specialized palliative home care | 79% | 1.14  (1.07-1.20) | 32% | 0.90  (0.84-0.95) | 81% | 2.24  (2.10-2.38) |
|  | Died elsewhere but had SPC^¥^ consultation for symptom management | 83% | 1.55  (1.42-1.70) | 44% | 1.51  (1.39-1.63) | 66% | 1.00  (0.92-1.09) |
| Colorectal cancer | No specialist palliative care involvement | 82% | ref | 34% | ref | 76% | ref |
|  | Died in a PC setting or received specialized palliative home care | 84% | 1.12  (1.03-1.20) | 34% | 0.86  (0.81-0.92) | 82% | 1.66  (1.54-1.79) |
|  | Died elsewhere but had SPC consultation for symptom management | 88% | 1.59  (1.41-1.78) | 44% | 1.40  (1.27-1.53) | 71% | 0.88  (0.80-0.97) |
| Prostate cancer | No specialist palliative care involvement | 80% | ref | 34% | ref | 73% | ref |
|  | Died in a PC setting or received specialized palliative home care | 85% | 1.21  (1.12-1.31) | 39% | 1.05  (0.98-1.13) | 78% | 1.54  (1.42-1.67) |
|  | Died elsewhere but had SPC consultation for symptom management | 89% | 1.85  (1.62-2.10) | 50% | 1.73 (1.58-1.91) | 69% | 0.88  (0.80-0.98) |
| Pancreatic cancer | No specialist palliative care involvement | 84% | ref | 38% | ref | 74% | ref |
|  | Died in a PC setting or received specialized palliative home care | 86% | 1.04  (0.95-1.15) | 37% | 0.85  (0.78-0.92) | 82% | 1.81  (1.66-1.98) |
|  | Died elsewhere but had SPC consultation for symptom management | 89% | 1.51  (1.30-1.75) | 48% | 1.39  (1.24-1.55) | 73% | 1.00  (0.89-1.12) |
| Hematological malignancy | No specialist palliative care involvement | 75% | ref | 30% | ref | 69% | ref |
|  | Died in a PC setting or received specialized palliative home care | 78% | 1.21  (1.12-1.31) | 31% | 0.99  (0.91-1.08) | 82% | 2.12  (1.93-2.33) |
|  | Died elsewhere but had SPC consultation for symptom management | 85% | 1.86  (1.60-2.17) | 43% | 1.66  (1.45-1.89) | 69% | 1.03  (0.90-1.18) |
| Breast cancer | No specialist palliative care involvement | 79% | ref | 32% | ref | 75% | ref |
|  | Died in a PC setting or received specialized palliative home care | 81% | 1.03  (0.94-1.14) | 31% | 0.82  (0.75-0.91) | 83% | 1.93  (1.73-2.14) |
|  | Died elsewhere but had SPC consultation for symptom management | 85% | 1.41  (1.21-1.64) | 40% | 1.28  (1.12-1.45) | 74% | 1.03  (0.89-1.18) |
| Urinary tract cancer | No specialist palliative care involvement | 81% | ref | 34% | ref | 73% | ref |
|  | Died in a PC setting or received specialized palliative home care | 85% | 1.24  (1.11-1.37) | 36% | 0.97  (0.88-1.07) | 81% | 1.73  (1.56-1.92) |
|  | Died elsewhere but had SPC consultation for symptom management | 88% | 1.69  (1.42-1.99) | 47% | 1.54  (1.35-1.75) | 67% | 0.82  (0.72-0.93) |
| Cancer of female genital organs | No specialist palliative care involvement | 83% | ref | 36% | ref | 74% | ref |
|  | Died in a PC setting or received specialized palliative home care | 83% | 0.94  (0.84-1.04) | 35% | 0.86  (0.77-0.94) | 81% | 1.69  (1.52-1.88) |
|  | Died elsewhere but had SPC consultation for symptom management | 87% | 1.33  (1.12-1.57) | 47% | 1.43  (1.26-1.63) | 72% | 1.02  (0.89-1.17) |
| Gastric or esophageal cancer | No specialist palliative care involvement | 79% | ref | 33% | ref | 73% | ref |
|  | Died in a PC setting or received specialized palliative home care | 82% | 1.10  (0.98-1.23) | 34% | 0.90  (0.80-1.00) | 81% | 1.73  (1.53-1.95) |
|  | Died elsewhere but had SPC consultation for symptom management | 85% | 1.45  (1.22-1.72) | 40% | 1.24  (1.06-1.43) | 71% | 0.92  (0.79-1.07) |
| Cancer of unknown primary | No specialist palliative care involvement | 80% | ref | 33% | ref | 71% | ref |
|  | Died in a PC setting or received specialized palliative home care | 84% | 1.28  (1.14-1.44) | 34% | 0.95  (0.85-1.06) | 81% | 1.92  (1.71-2.16) |
|  | Died elsewhere but had SPC consultation for symptom management | 86% | 1.45  (1.22-1.72) | 43% | 1.41  (1.22-1.63) | 70% | 1.00  (0.86-1.16) |
| Liver cancer | No specialist palliative care involvement | 82% | ref | 36% | ref | 72% | ref |
|  | Died in a PC setting or received specialized palliative home care | 84% | 1.04  (0.91-1.20) | 32% | 0.79  (0.69-0.90) | 82% | 1.92  (1.67-2.20) |
|  | Died elsewhere but had SPC consultation for symptom management | 87% | 1.37  (1.09-1.71) | 42% | 1.30  (1.07-1.56) | 72% | 1.03  (0.85-1.24) |
| Cancer of the brain or CNS | No specialist palliative care involvement | 72% | ref | 20% | ref | 79% | ref |
|  | Died in a PC setting or received specialized palliative home care | 70% | 0.90  (0.79-1.02) | 19% | 0.94  (0.79-1.12) | 88% | 1.98  (1.64-2.40) |
|  | Died elsewhere but had SPC consultation for symptom management | 78% | 1.41  (1.17-1.70) | 28% | 1.55  (1.26-1.91) | 81% | 1.12  (0.88-1.40) |
| Skin cancer | No specialist palliative care involvement | 79% | ref | 32% | ref | 75% | ref |
|  | Died in a PC setting or received specialized palliative home care | 79% | 1.03  (0.90-1.19) | 31% | 0.85  (0.73-0.98) | 82% | 1.56  (1.33-1.83) |
|  | Died elsewhere but had SPC consultation for symptom management | 86% | 1.66  (1.31-2.09) | 39% | 1.30  (1.07-1.58) | 73% | 0.92  (0.74-1.13) |
| Cancer of the galbladder or biliary tract | No specialist palliative care involvement | 83% | ref | 33% | ref | 77% | ref |
|  | Died in a PC setting or received specialized palliative home care | 84% | 1.00  (0.84-1.20) | 32% | 0.89  (0.75-1.04) | 83% | 1.68  (1.41-2.01) |
|  | Died elsewhere but had SPC consultation for symptom management | 88% | 1.38  (1.06-1.81) | 43% | 1.49  (1.20-1.84) | 74% | 0.91  (0.72-1.14) |
| Cancer of the head and neck region | No specialist palliative care involvement | 78% | ref | 29% | ref | 72% | ref |
|  | Died in a PC setting or received specialized palliative home care | 80% | 1.09  (0.92-1.29) | 31% | 1.01  (0.85-1.20) | 82% | 1.93  (1.60-2.32) |
|  | Died elsewhere but had SPC consultation for symptom management | 84% | 1.47  (1.12-1.91) | 39% | 1.48  (1.17-1.89) | 69% | 0.88  (0.69-1.13) |
| Cancer of the bone or soft tissue | No specialist palliative care involvement | 85% | ref | 39% | ref | 66% | ref |
|  | Died in a PC setting or received specialized palliative home care | 84% | 0.94  (0.73-1.23) | 39% | 0.89  (0.71-1.12) | 78% | 2.07  (1.64-2.62) |
|  | Died elsewhere but had SPC consultation for symptom management | 87% | 1.17  (0.79-1.73) | 50% | 1.51  (1.10-2.06) | 64% | 0.96  (0.71-1.31) |
| Other | No specialist palliative care involvement | 81% | ref | 34% | ref | 75% | ref |
|  | Died in a PC setting or received specialized palliative home care | 81% | 0.95  (0.83-1.09) | 33% | 0.82  (0.72-0.93) | 80% | 1.60  (1.39-1.84) |
|  | Died elsewhere but had SPC consultation for symptom management | 86% | 1.44  (1.16-1.79) | 44% | 1.37  (1.15-1.64) | 72% | 0.95  (0.78-1.15) |

*odds ratios were adjusted for age and sex

± PC (palliative care) setting includes hospice and inpatient palliative care

¥ SPC = specialist palliative care

*
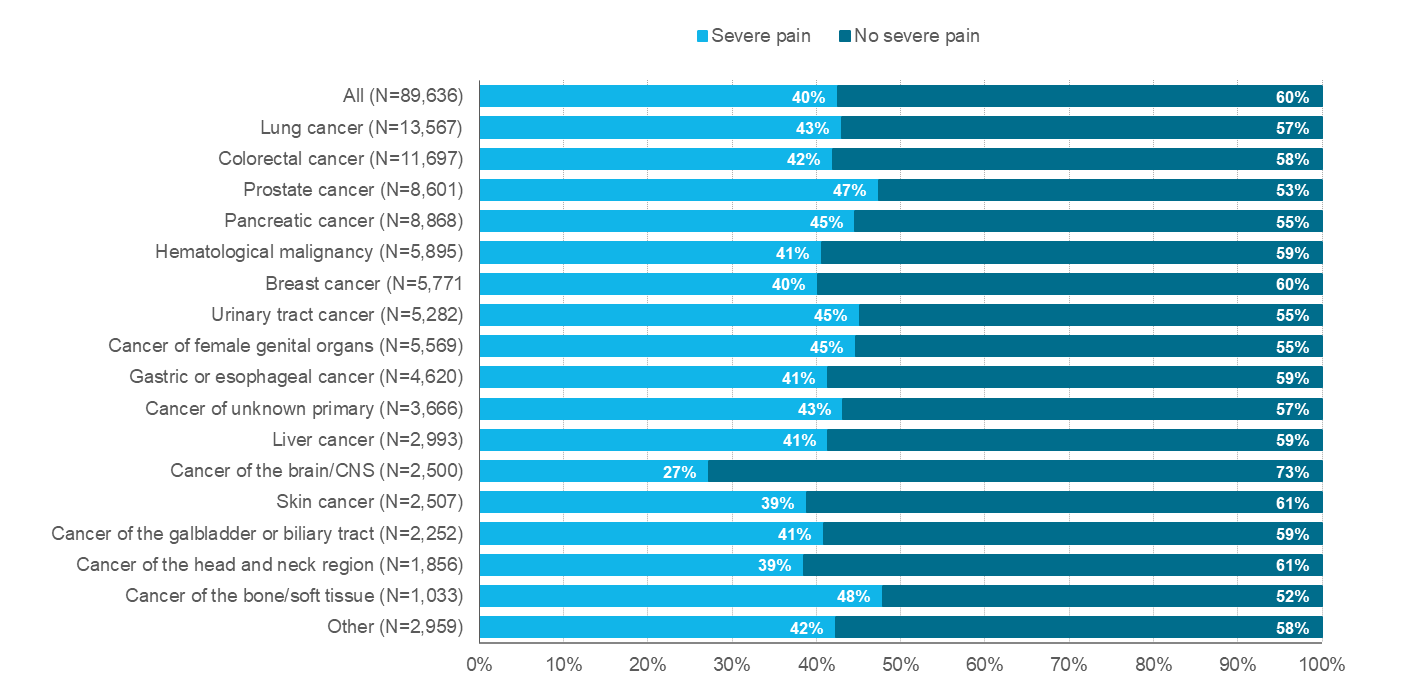
*

*Supplementary figure 1: Prevalence (%) of severe pain during the last week of life in patients with cancer in whom it was reported that their pain was assessed using validated tools.*
